# Hyperopia: a practical introduction

**Published:** 2024-05-15

**Authors:** Sonia Mavi, Jessica Massie, Ving Fai Chan, Priya Morjaria

**Affiliations:** 1 PhD student and Optometrist: Queens University Belfast, Royal Victoria Hospital, Belfast, Northern Ireland, UK.; 2 Freelance Global Eye Health Consultant and Public Health Optometrist, Australia.; 3 Senior Lecturer and Public Health Optometrist: Queens University Belfast, Royal Victoria Hospital, Belfast, Northern Ireland, UK.; 4 Assistant Professor and Public Health Optometrist: London School of Hygiene & Tropical Medicine and Head of Global Programme Design: Peek Vision, UK.

**Hyperopia is a common eye condition in children that affects near vision. Detecting and treating it in time can reduce the risk of squint and amblyopia.**

**Figure 1:**
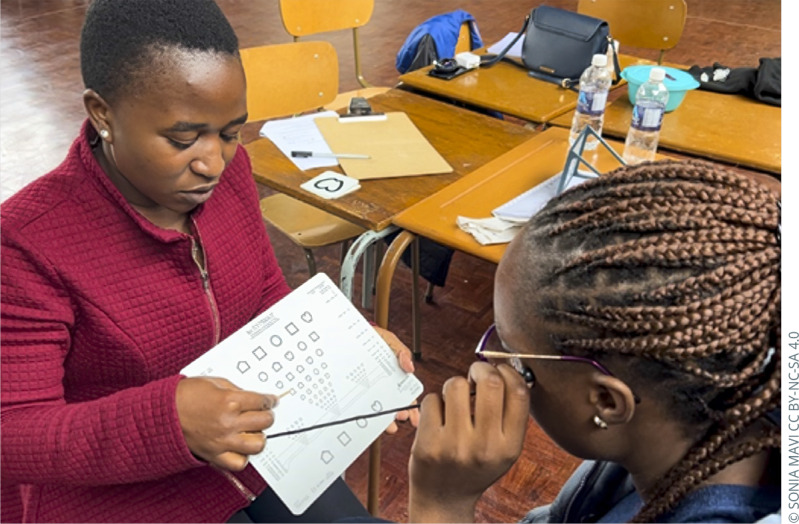
Measuring near visual acuity with a conventional LogMAR near vision chart using Lea symbols at 40 cm. zimbabwe

Hyperopia, also called farsightedness, is a common problem in children worldwide. The limited data available suggest that around 4.6% of children, on average, have clinically significant hyperopia (defined as a spherical equivalent of ≥+2.00 D), ranging from 2.2% in Southeast Asia to 14.3% in the Americas.^1^

Hyperopia affects the eye's ability to focus, especially at near distances. This happens when the eyeball is too short, or the cornea is not curved enough, causing the rays of light to focus behind the retina instead of on it. Babies are usually born moderately hyperopic, but this gradually decreases as their eyes grow and develop: from an average spherical equivalent of +2.00 D at age 3 months, to just over +1.00 D by the time they are 3 or 4 years old.^2^,^3^

Children with adequate focusing ability (accommodation) can often overcome uncorrected hyperopia, including clinically significant hyperopia (defined as ≥+2.00 D). When these children start attending school, however, the need to accommodate for longer periods of time can lead to eye strain, headaches, and blurred vision, which can affect their reading and other school-related tasks. Children who have higher levels of hyperopia that cannot be overcome using their accommodative ability experience poorer near vision and near depth perception, which also affects their academic performance.^4^ Some children may even experience poorer distance vision due to hyperopia.

Unless their hyperopia is corrected by the age of 7 years, children with clinically significant uncorrected hyperopia (defined as ≥+2.00 D) are also at risk of developing strabismus and amblyopia, resulting in irreversible visual impairment in the affected eye.^5^,^6^

## Screening for hyperopia

Because screening for hyperopia is challenging, children with clinically significant hyperopia are often not identified or treated. At present, the distance visual acuity (VA) test is the main method used in school-based programmes in low- and middle-income countries to identify children with reduced vision. This test is useful in detecting myopia, but not always hyperopia, as only some children with clinically significant hyperopia experience reduced distance vision.

Given the fact that the majority of a child's visual development is thought to happen by the age of 7 years, it is important to detect and treat clinically significant hyperopia as early as possible. However, in settings with limited resources, it is usually recommended to do this when children start school.

There is no globally accepted consensus on how to screen children for hyperopia. We recommend using either the **plus-lens test** or the **near visual acuity test** to screen for significant hyperopia, but only if there are enough resources and well-trained personnel to carry out the tests.

## 1. The plus lens test

The plus lens test involves putting a +2.50 D lens in front of the eye and measuring the difference (if any) in distance visual acuity.

Because children with hyperopia can see more clearly through a plus lens, especially at near distances, the addition of a plus lens should not affect their distance visual acuity very much (Figure 2a). Children who do not have hyperopia, however, will experience significant blurring of their vision when looking through the same lens (Figure 2b).

To distinguish between children with and without clinically significant hyperopia, the criteria for failing or passing the test are as follows:

**Fail (child has hyperopia):** The distance visual acuity while looking through +2.50 D lens worsens by **two lines** **or less** compared to the unaided distance visual acuity.

**Pass (no hyperopia):** The distance visual acuity while looking through the +2.50 D lens worsens by **more than** **two lines** compared to the unaided distance visual acuity.

### How to perform the plus lens test

1. After completing the unaided distance visual acuity test, cover the left eye and place a +2.50 D lens over the child's right eye.
2. Direct the child's attention to the distance vision chart and check their visual acuity again, by asking them to identify the letters or symbols.
3. Decide whether the child has passed or failed, using the criteria above.
4. Repeat for the other eye.

## 2. The near visual acuity test

The near visual acuity test involves measuring the near distance vision. To distinguish between children with and without clinically significant hyperopia, the criteria for failing or passing the near visual acuity test are as follows:

**Fail (child has hyperopia):** If the child is unable to correctly identify 3 out of 5 letters or symbols on the 0.2 LogMAR (or 6/9.5 Snellen) line.

**Pass (no hyperopia):** If the child is able to correctly identify 3 out of 5 letters or symbols on the 0.2 LogMAR (or 6/9.5 Snellen) line.

The steps are as follows:

1. Use a near visual acuity chart, such as a Sloan letter or Lea symbol chart; the latter is helpful for children who are not comfortable with the alphabet.
2. Hold the near visual acuity chart at 40 cm from the face, at eye level (Figure 1). Ensure that the child is not leaning forward.
3. Cover the left eye with an occluder or patch and test the right eye.
4. Ask the child to identify the letters or symbols on each line, as directed.
5. Decide whether the child has passed or failed the test, using the criteria mentioned earlier.
6. Repeat for the other eye.

## Prescribing for hyperopia

To determine the amount of hyperopia, it is advisable to conduct a cycloplegic refraction whenever possible, as it yields reliable measurements (see article on cycloplegic refraction in this issue).

When prescribing spectacles for hyperopic children, the aim is to decrease the demand for focusing (accommodative demand) and provide clear and comfortable vision with both eyes. Therefore, eye care providers should prescribe the maximum plus power that children can comfortably accept while still maintaining clear vision. Factors to consider include the child's age, the amount of hyperopia in each eye, and the presence of strabismus, among others. In most cases, children are better able to tolerate a lower plus prescription, except when there is an inward eye turn (esotropia). In such cases, the full cycloplegic correction is required to minimise or eliminate the squint.

“To determine the amount of hyperopia, it is advisable to conduct a cycloplegic refraction whenever possible.”

It is also important to recognise that children with anisometropia (a difference in refractive error between the eyes) of +1.00 D or greater, or who have a similar level of hyperopia (isometropia) of +5.00 D or greater in both eyes, are at risk of hyperopic refractive amblyopia.^7^,^8^ The American Academy of Ophthalmology has practical guidelines for these children, according to age and other factors (see Table 1).

**Figure 2a:**
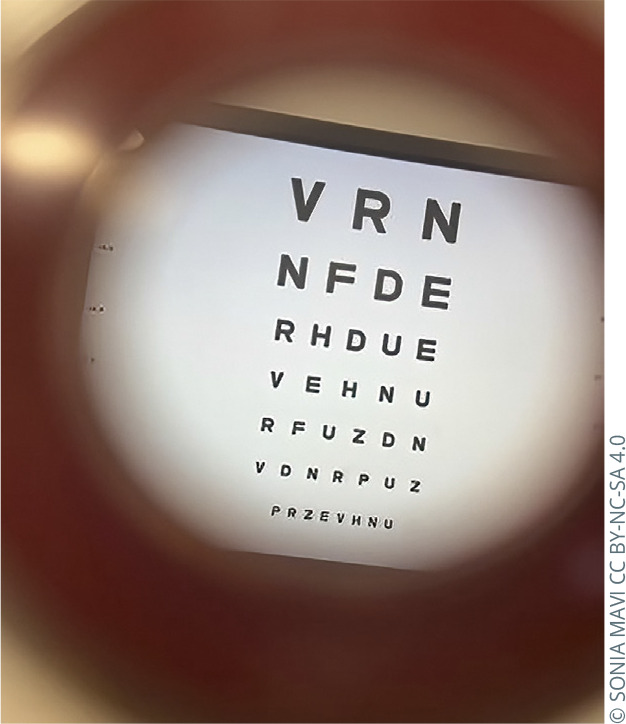
Children with hyperopia will not experience significant blurring of their distance vision when looking through a +2.50 D lens.

**Figure 2b:**
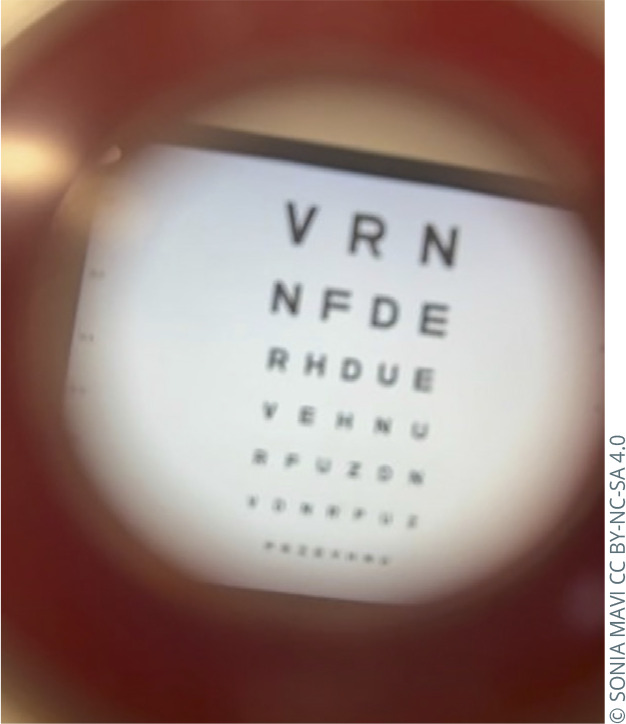
A child who does not have hyperopia will experience significant blurring of their vision when looking through a +2.50 D lens.

**Table 1:** The American Academy of Ophthalmology's recommended prescribing guidelines for hyperopia. Available at tinyurl.com/AAOhyperopia

For children with isometropia (similar level of refractive error in both eyes)				
**Refractive status**	**Age <** **1 year**	**Age 1 to <** **2 years**	**Age 2 to <** **3 years**	**Age 3 to <** **4 years**
Hyperopic isometropia (without esotropia)	≥ + 5.00 D	≥ + 5.00 D	≥ + 4.50 D	≥ + 3.50 D
Hyperopic isometropia (with esotropia)	≥ + 1.50 D	≥ + 1.00 D	≥ + 1.00 D	≥ + 1.00 D

(Source: **AAO Prescribing guidelines for hyperopia**)

**Table:** 

For children with anisometropia (an unequal level of refractive error between the eyes)				
**Refractive status**	**Age <** **1 year**	**Age 1 to <** **2 years**	**Age 2 to <** **3 years**	**Age 3 to <** **4 years**
Hyperopic anisometropia (without esotropia)	≥ + 2.50 D	≥ + 2.00 D	≥ + 1.50 D	≥ + 1.50 D

(Source: **AAO Prescribing guidelines for hyperopia**)
